# Adapting Agriculture to Climate Change: Are Climate‐Smart Practices Important in Burkina Faso?

**DOI:** 10.1002/pei3.70113

**Published:** 2025-12-30

**Authors:** Benoit Bado, Noël Thiombiano, Nestor Tiehi Tito

**Affiliations:** ^1^ Department of Economics, Center for Economic and Social Studies, Documentation and Research, CEDRES University of Thomas Sankara Ouagadougou Burkina Faso; ^2^ Department of Economics, Economic and Social Studies, Documentation and Research Center (CEDRES) Thomas SANKARA University Ouagadougou Burkina Faso; ^3^ Economics Department University of Cocody Abidjan Côte d'Ivoire

**Keywords:** agricultural resilience, Burkina Faso, climate change, climate‐smart practices, multivariate probit

## Abstract

In Burkina Faso, smallholder farmers rely heavily on rain‐fed agriculture, which is affected by climate change. The adoption of climate‐smart practices is essential to strengthen the resilience of agricultural systems to climate change and improve household food security and, consequently, global food security. Despite the great potential of these practices to combat the effects of climate change on agriculture, their adoption by farmers remains low or limited. The reasons for this low adoption are varied, suggesting that the factors are largely contextual. This research analyzes the determinants of the adoption of climate‐smart practices among farmers in Burkina Faso in the context of innovation diffusion. To do this, a multivariate probit regression model was used on survey data from 48,159 plots owned by farmers in the country. The results show that age, gender, access to credit, access to extension services, property rights, livestock ownership, and education are the main determinants of the adoption of climate‐smart practices in Burkina Faso. Large‐scale awareness‐raising, training, and promotion, while promoting access to credit and land ownership documents, are necessary for better adoption of climate‐smart practices.

## 1 Introduction

Agriculture remains a vital sector of the economy in most African countries, and its development has significant implications for food security and poverty reduction. In Burkina Faso, agriculture remains one of the driving forces of economic development. This sector provides a living for over 87% of the working population. Nearly 80% of jobs come from this sector; agricultural GDP accounts for 35% of total GDP, whereas 57% of export earnings come from agricultural products (Zidouemba and Gerard 2018). However, its rain‐fed or subsistence agricultural production system is limited by multiple and complex biophysical challenges, such as climate change and variability, low soil fertility, and the prevalence of pests and diseases (Shiferaw et al. 2014). In Burkina Faso, climate change, through its harmful effects, severely affects the income of agricultural households, the availability of agricultural products and, in general, food security (Sanfo and Gérard 2012). Faced with this dynamic phenomenon of unpredictable magnitude, adaptation is an option that would significantly reduce the negative impact of climate. To maintain the livelihoods of farming households despite the agricultural production climate, climate‐smart agriculture (CSA) has emerged as a very promising approach that can integrate the benefits of a sustainable increase in agricultural productivity, adaptation and building resilient agricultural and food security systems, as well as reducing greenhouse gas (GHG) emissions from agricultural activities (FAO 2010).

Given the current context in Burkina Faso, the ASC presents itself as a very important strategy. Indeed, Mkwambisi et al. (2016) estimated that the adoption of CSA leads to an increase in maize yield between 9% and 37% and an increase in crop income between 36% and 60%, depending on the CSA strategy. Moreover, if the implementation of these climate‐smart technologies (CSAs) is successful, it could stimulate overall economic growth through cross‐sectoral linkages while preserving natural resources (Sanchez et al. 2009). However, various agricultural technologies and practices labeled as climate smart are not effective because they could have resulted in a low adoption rate by farmers in developing countries (Westermann et al. 2018).

Researchers have shown that the choices and use of these climate change adaptation strategies by farmers depend on a number of socioeconomic, institutional and biophysical factors (Bryan et al. 2013). For example, the results of previous empirical studies have shown that gender, insufficient information, limited agricultural awareness and experience, household size, years of education, access to credit facilities, access to extension services, and nonfarm income‐generating activities are all factors that hinder adaptation to climate change (Gebru et al. 2020). Other studies have analyzed the factors that explain the intensity of adoption of climate adaptation strategies. This is the case for Mwaura et al. (2021), Asule et al. (2024) in Kenya, Ng'ombe et al. (2024) in Malawi. Their work shows that the main determinants were socio‐economic factors (age, gender, education, membership of an organization), biophysical factors (plot size, soil fertility), behavioral factors (perception), and institutional factors (access to extension services, access to credit). Empirical studies conducted in Burkina Faso have also indicated that the level of education, livestock ownership and credit availability significantly influence the adaptive capacity of Burkinabe farmers (Thinda et al. 2020; Damba et al. 2018; Yameogo et al. 2017). However, the adaptation of rural households to climate change is not unique, and the factors that determine their use differ from one area to another. In view of the above, one can ask the following questions: What are the factors that promote or limit the adoption of climate‐smart practices among farmers in Burkina Faso?

The objective of this work is to analyze the factors that promote or limit the adoption of climate‐smart practices among farmers in Burkina Faso.

Despite the abundant literature on climate change adaptation strategies, most empirical studies (Thinda et al. 2020) have focused on the adoption of practices taken individually and independently but also in specific areas. Additionally, these studies have often adopted conventional estimation methods based on logit or probit models. The few empirical studies that have focused on more than one adaptation strategy at a time have also adopted a multinomial logit as an analytical approach (Al‐Amin et al. 2019; Damba et al. 2018; Yameogo et al. 2017), thus basing themselves on the mutual exclusivity of alternatives. However, the adoption of any adaptation strategy could lead to the use of another strategy (Wainaina et al. 2016; Kassie et al. 2013). Therefore, the adoption of a random adaptation strategy is not (in total) independent of other strategies and should be analyzed as such. This work is therefore intended as a contribution at this level while carrying out the analysis across the entire country.

The remainder of the work is structured into four sections. The first section reviews the results of empirical work on the various factors that determine the adoption of climate‐smart practices. The second section addresses the methodology used. The third section discusses the results obtained, and the last section is devoted to the conclusion and the resulting economic policy implications.

## 2 Empirical Review of Different Types of Climate‐Smart Adaptation Strategies and the Factors Influencing Their Adoption

Most studies on the adoption of climate change adaptation strategies emphasize household socioeconomic characteristics as well as institutional and agroecological factors as factors influencing household choice. The effects of these factors are positive for some and negative for others.

In Pakistan, Shahzad and Abdulai (2021) reported that being informed/trained and having access to credit are the strongest indicators of the potential adoption of climate‐smart agriculture (CSA). They reported that CSA practices significantly reduce food insecurity and poverty.

Adhikari (2018) in Nepal highlighted that the adoption of climate‐smart practices by producers faced natural limits (ecological and physical) due to particular environmental endowments; knowledge, technology and financial constraints; and social barriers (cognitive, normative and institutional). In Ethiopia, Tazeze et al. (2012) analyzed the determinants of farmers' choice of climate change adaptation strategies. Using a multinomial logit analysis, they concluded that the gender of the household head, the age of the household head, the level of education, the size of the family, the ownership of livestock, the agricultural and nonagricultural income of the household, access to credit, distance from the market center, access to agricultural extension, agroecological zones, access to climate information and contact with extension have significant impacts on climate change adaptation strategies.

In Burkina Faso, Maré et al. (2022) identified factors affecting sustainable agricultural intensification by farmers. Using a negative binomial regression model on survey data from 1898 rural households in the country, they concluded that land tenure and access to credit are the main determinants of sustainable agricultural intensification in Burkina Faso. In South Africa, Setshedi and Modirwa (2020) surveyed 170 smallholder farmers (livestock, cereals, and mixed crops) about climate‐smart agricultural practices implemented to address climate change. They reported that the main barrier to adopting climate‐smart practices was a lack of knowledge and information. Most rural farmers have poor internet connectivity, are less educated, and lack sufficient financial resources.

For their part, Makate et al. (2018), in their work carried out in different agroecological zones of Malawi, Mozambique, Zambia, and Zimbabwe, demonstrated that farm size and financial circumstances play important roles in the adoption of CSA.

Musafiri et al. (2022) used multivariate and ordered probit models to show that in Kenya, gender, level of education, age of the head of household, family size, contact with extension agents, access to weather information, amount of arable land, livestock owned, perception of climate change, soil fertility, and persistent soil erosion influence the adoption of climate‐smart practices (CSPs). The ordered probit model revealed that gender, arable land, livestock owned, soil fertility, and persistent soil erosion were determining factors in the adoption of sustainable agricultural practices. In addition, they led to complementarities and substitutions between climate‐smart practices, meaning that certain practices should not be adopted simultaneously. Also in Kenya, Asule et al. (2024) assessed the determinants of simultaneous awareness and adoption of climate‐resilient practices (CRPs), as well as their intensity, in the Central Highlands of the country. Using a multivariate probit model and Poisson regression on cross‐sectional data collected from 400 smallholder farmers, the authors conclude that awareness and adoption of specific CCPs, as well as their intensity, were determined by occupation, age, farming experience, household size, soil fertility management, climate change adaptation, agricultural training, and geographical location. They also showed that agricultural training for smallholders was an important determinant of awareness, level of adoption, and intensity of CSPs.

Shani et al. (2024) in Malawi found that despite the high potential of climate‐smart practices to combat the effects of climate change, their adoption by smallholders remains low. Using a convergent mixed research methodology and data collected through questionnaires, interviews, and grids, they conclude that compatibility and simplicity are the main determinants of the adoption of these practices. Furthermore, they emphasize that climate‐smart agricultural practices, which require significant labor and capital, are less likely to be adopted by these smallholders. Following the same logic and in the same area, Ng'ombe et al. (2024) analyzed the factors that influence participation in agricultural projects, as well as the level and intensity of adoption of the promoted greening practices. To achieve their goal, data were collected from 244 households participating in the SAPMaRT project in two districts: Kasungu and Mzimba. Using binary probit and Poisson regression models, they show that participation in projects has a positive and significant effect on the level and intensity of adoption of these practices. In addition, the level of education, household size, climate awareness, and agricultural extension services have a positive influence on participation in the projects. Similarly, participation in the project, level of education, age, and agricultural extension services positively influenced the adoption of good agricultural practices. On the other hand, the age of the head of household had a negative impact on this adoption. In terms of the intensity of adoption, the head of household's participation in the project, their level of education, the size of their land, and agricultural extension services were found to be the main determining factors.

In Mali Diaby et al. (2020) reported that assisted natural regeneration (RNA) is an adaptation strategy that can effectively increase the resilience of farms to climatic hazards. Using a logit model to analyze the determinants of RNA adoption in the districts of Diéma and Kolokani, they concluded that farmers' training in RNA, harvesting, fertilizer use, perceptions of field fertility, and income lead to an increase in the probability of adopting RNA.

In light of the literature, farmers respond to the adverse effects of climate change by adopting a number of agricultural practices. The factors influencing the adoption of these climate‐smart practices and technologies are summarized as sociodemographic factors, institutional factors, farm characteristics, economic factors, technology characteristics and information transmission system characteristics.

## 3 Conceptual Framework of the Study Model and Empirical Specification of the Model

This section presents the theoretical framework of our work, the empirical model, and the estimation method.

### 3.1 Analysis Model

The analytical approaches that are commonly used in adoption decision studies involving multiple choices are the multinomial logit (MNL) and multinomial probit (MNP) models. These two models are very important for analyzing farmers' adaptation decisions because they are generally adopted jointly and have many advantages. However, these methods have a very large limitation. Indeed, according to these models, individuals make a single choice each time, that is, the one that provides them with maximum utility. These models are based on the assumption that the individual (consumer or producer) makes a single choice from a set of global choices (Walsh 1995; Dubé 2004). This is both a theoretical and empirical limitation, as reality shows that this assumption is often unrealistic and only serves to artificially restrict the set of choices available to the individual. To overcome this shortcoming, multivariate logit (MVL) and multivariate probit (MVP) models have been developed to analyze multiple choices.

The adoption of any new technology, such as a climate change adaptation strategy, remains an individual decision (Rogers 2003). This decision is generally based on the economic principle of rationality of neoclassical theory (Varian 2008). Thus, the producer adopts a new technology if the latter allows him to maximize his utility. Let us suppose a producer i who seeks to maximize his utility linked to a production system integrating a given climate change adaptation strategy (π). The producer will adopt a given climate‐smart practice if the expected utility, represented by $U_{1}^{*}\left(\pi \right)$, is greater than that which he would derive if he had not adopted it, represented by $U_{0}^{*}\left(\pi \right)$, or if $U_{1}^{*}\left(\pi \right)$ > $U_{0}^{*}\left(\pi \right)$. The utility to be maximized $U_{i}$ and on which the producer's decision to adopt or not is based is not observable but generally depends on a set of socioeconomic, demographic and institutional factors $X_{i}$ and can therefore be represented by the latent variable as follows:

(1) $$U_{i}^{*}=X_{i}\beta +\varepsilon_{i},\;i=1,2,3\ldots N$$

With *β* as the parameter vector, $\varepsilon_{i}$ to be estimated is a random disturbance.

This research focused on four climate‐smart practices (CSAs), namely, improved seed varieties, organic manure, water and soil conservation (WSC) techniques and agroforestry. Therefore, and as suggested by the empirical literature on the adoption of agricultural technologies (Kassie et al. 2013; Wainaina et al. 2016) the producer will adopt all the CSA practices that contribute to maximizing his expected utility, thus stipulating an interdependence of the decisions to adopt each of these practices. In other words, the decision to adopt the CSA practice for climate change j by producer i is correlated with the decision to adopt practice k. Thus, it is imperative to use a model capable of simultaneously estimating the determinants of the different CSA practices. To address all this, the literature suggests the use of a multivariate probit regression model (Timu et al. 2014). Following Adekambi and Hinnou (2020) in Benin, Musafiri et al. (2022) in Kenya, and Ng'ombe et al. (2024) in Malawi, we adopted a multivariate probit model in our work to analyze the determinants of the adoption of climate‐smart practices among producers in Burkina Faso. The correlation of error terms where a positive sign represents complements where a negative sign indicates substitutes between different CSAs (Mulwa et al. 2017; Oyetunde‐Usman et al. 2021) Joint adoptions for the four climate‐smart practices can be modeled by a system of four dichotomous adoption equations as follows:

(2) $$\left\{\begin{array}{c} Y_{1}=1\;\text{if}\;U_{1}^{*}>U_{0\;}^{*},Y_{1}=0\;\text{if otherwise}\\ Y_{2}=1\;\text{if}\;U_{2}^{*}>U_{0\;}^{*}Y_{2}=0\;\text{if otherwise}\\ Y_{3}=1\;\text{if}\;U_{3}^{*}>U_{0\;}^{*}Y_{3}=0\;\text{if otherwise}\\ Y_{4}=1\;\text{if}\;U_{4}^{*}>U_{0\;}^{*}Y_{4}=0\;\text{if otherwise} \end{array}\right.$$

The probability of adoption of CSA climate practices (Equation 2) is estimated via the multivariate probit (MVP) regression model to account for possible correlations between the error terms of the different binary adoption equations (Greene 2008). The model estimated with the variables included in the estimates is presented as follows:

(3) $$\begin{array}{cc} {\mathrm{CSA}}_{j} & =\alpha_{0}+\alpha_{1}\mathrm{Age}+\alpha_{2}{\text{Farmers}}^{\prime }\;\text{organization}+\alpha_{3}\text{Gender}\\ & +\alpha_{4}\text{Education}+\alpha_{5}\text{Access to credit}+\alpha_{6\;}\text{Livestock ownership}\\ & +\alpha_{7}\text{Extension services}+\alpha_{8}\text{Relief of the plot}\\ & +\alpha_{9}\text{Property rights}+\alpha_{11}\text{Area}+{ԑ}_{\mathrm{it}} \end{array}$$

The dependent variable ${\mathrm{CSA}}_{j}$ is a dichotomous variable that takes a value of 1 if producer i adopts the CSA strategy j (with j = Improved seed variety, organic manure, water and soil conservation (CES) techniques and agroforestry) and 0 otherwise.

### 3.2 Description of Variables and Data Sources

This subsection describes the variables used in this research and provides the source of the data. The choice of variables used is based on theoretical and empirical literature relating to the analysis of the adoption of sustainable agricultural practices, as well as on data availability. These variables are summarized in Table 1 below.

**TABLE 1:** Summary of the explanatory variables defined in the research.

Explanatory variables	Measure	Sign
Age of head of household	In number of years	+/−
Gender	1 if male, 0 if female	+/−
Education	1 if he has received formal education, 0 if not	+
Surface area	In hectares	+/−
Property rights	1 if he has a formal document, 0 if not	+
Access to credit	1 if it accesses, 0 if not	+
Plot relief	1 if plain/plateau; 2 if lowland; 3 if slope	+/−
Farmer's organization	1 if it belongs, 0 if not	+
Livestock ownership	1 if it has, 0 if not	+
Extension service	1 if it accesses, 0 if not	+

*Note:* *Source*: Author.

#### 3.2.1 Dependent Variable

In our work, the dependent variable included four climate‐smart practices: water and soil conservation (CES) techniques; improved seeds; agroforestry; and organic manure. These WSC practices include integrated soil fertility management (ISFM) techniques and soil management technology. According to Nana (2015), on the one hand, GIFS techniques and soil management technology, as well as their combinations, contribute to reducing the damage caused by climate change to the agricultural sector in terms of increasing yields.

Khaitov et al. (2019) emphasize that organic manure is considered climate‐smart because it improves soil structure and water retention capacity with minimal leaching, reducing the need for synthetic fertilizers and associated greenhouse gas emissions. Improved seeds are promising, stress‐tolerant seeds that have the ability to withstand abiotic stress such as drought. They increase production per unit area, reduce production costs, and maintain underground biomass during periods of drought, as evidenced by Masuka et al. (2017). Water and soil conservation techniques (WSC) aim to make efficient use of water resources and soil nutrients by managing water runoff to prevent the leaching of soil nutrients, controlling water availability due to possible pockets of drought linked to poor rainfall distribution, and promoting soil permeability. Agroforestry is the most widespread farming system in Africa and consists of maintaining trees scattered throughout plots and cultivating between the trees. In addition, the trees found there have multiple uses for rural populations: wood, food, medicine, fiber, fodder, resin, latex, tannin, etc. The leaves, wood, and fruit are used, as well as the roots, branches, and flowers. In these plots, trees protect the soil from erosion, improve its fertility, provide shade for plants that cannot tolerate full sun, reduce the harmful effects of wind, concentrate moisture, and sometimes serve as shade for rest. They are also a symbol of social status and help to visualize plot boundaries and mark property lines.

The modalities are defined as follows for each of the different CSA practices (dependent variable): 0 if farmers do not adopt the practice in question and 1 if they do.

#### 3.2.2 Explanatory Variables

The choice of explanatory variables is based on the availability of data and the literature. Therefore, the explanatory variables retained in the framework of this research include household characteristics such as the age of the head of household, the sex of the head of household, possession of livestock, the level of education, and property rights.

Farm characteristics include land area, distance from the farm to the market, and terrain. Institutional factors include access to credit and access to extension services (Hassan and Nhemachena 2008). All the explanatory variables are listed in Table 1 below.

##### 3.2.2.1 Socioeconomic Factors

**Age of the household head**: Age can have a positive or negative effect (or a threshold effect) on the use of climate‐smart practices. The older a farmer is, the more agricultural experience they have and the more exposed they are to past and present climate conditions. Conversely, younger farmers may have long‐term plans for agricultural investments in technologies whose benefits materialize gradually. According toWassie and Pauline (2018), older farmers are adopting climate‐friendly farming practices because they have more experience with farming and past and present climate conditions. However, Teklewold et al. (2019) noted that an increase in the age of the household head reduces the possibility of choosing and using climate‐smart agricultural practices because as a farmer ages, he or she tends to minimize activities that require a greater share of his or her labor and management activities than younger farmers do.

**The gender of the head of household**: This is a binary variable that takes a value of 0 if the household head is a woman and 1 if the household head is a man. Several studies have shown that gender is an important factor influencing adoption decisions at the farm level. In developing countries, women generally face an overload of household tasks more often than men do. Therefore, they may lack the time to seek information from extension services about climate‐smart agricultural practices and choose those that are suitable for their production. Compared with female household heads, households headed by men may have better access to information than those headed by women do, which helps them choose climate‐smart agricultural practices, which are essential for their production. A study conducted by Kifle (2021) revealed that male‐headed households are more likely to have access to climate change‐related technologies and information than female‐headed households. In contrast, gender is also mentioned in the literature as a factor that can influence the adoption of climate‐smart strategies, but with a mixed effect (Damba et al. 2018).

**Education**: This factor is very influential in adoption decisions. The more educated a producer is, the more information he or she has to assess the innovation better and therefore limit the level of uncertainty (Roussy et al. 2015). More educated and experienced farmers are expected to have knowledge and information about climate change and the practices they can use in response (Maddison 2007). The effect of these two variables on the use of climate change adaptation strategies is therefore positive.

**Ownership of livestock**: Livestock is considered a source of income, food, draught power, and an asset that indicates household wealth, which can increase the availability of capital and farmers' ability to invest in climate‐smart agricultural practices. Indeed, livestock size is an indicator of economic security. According to Amole and Ayantunde (2016), if a farmer has a large number of livestock, he is not threatened by the implementation of climate‐smart agricultural practices because he has full confidence in taking the risk related to climate change in his agricultural production by replacing the income from his livestock.

##### 3.2.2.2 Characteristics of the Operation

**The surface area**: the influence of this variable can be negative in the case of a technology adoption study. In rural areas, the majority of agricultural households are poor, so producers with more land will have difficulty adopting climate change adaptation strategies because of their requirements in terms of financial resources, labor, and agricultural equipment (Adeoti et al. 2003). However, Arodokoun et al. (2012) reported that increasing the area increases the probability of adopting strategies. The expected sign is mixed.

**The relief of the plot** is a categorical qualitative variable that takes a value of 1 if the plot is located on plain or plateau relief, 2 if the plot is located in a lowland, and 3 if the plot is on slope relief. This variable is likely to have a positive or negative effect on the probability of using climate‐smart practices. For the interpretation of this variable, we use the value 1, that is, plot located on a plain or plateau relief as a reference.

**Property rights**: The literature reports a positive theoretical link between property rights and the adoption of agricultural technologies. Indeed, owner‐producers are more willing to invest sustainably in their farms than nonowners, who fear recovery by the former after significant investments (Maré, 2022). In Burkina Faso, Eugenie et al. (2022) reported that formal property rights positively influence the likelihood of the adoption of stone bunds, half‐moons, and live hedges.

##### 3.2.2.3 Institutional Factors

**Access to credit:** Agricultural credit improves a farmer's financial ability to purchase inputs, thereby facilitating the implementation of climate‐smart agricultural practices. With more financial resources, farmers are able to use any information at their disposal to adapt their management practices according to the demands of climatic and other conditions. Similarly, farmers' access to credit simplifies cash flow constraints and allows them to purchase agricultural inputs such as improved seeds, fertilizers, chemicals, livestock feed, and farm equipment. Combary (2013); Sanou et al. (2017) for example, access to credit has a significant positive effect on the probability of technology adoption.

**Access to extension services**: Access to extension refers to the services provided to farmers by development agents regarding climate‐smart agricultural practices. Extension services play an important role in increasing awareness of climate‐smart agricultural practices and their use. This implies that farmers with greater access to information and technical support related to climate‐smart agricultural practices may be aware of the impacts of climate change and have already implemented or are more likely to implement climate‐smart agricultural practices (Belay et al. 2023).

**Participation in a farmer's organization** (OP): This qualitative variable is binary and takes a value of 1 if the individual belongs to a group and 0 if not. In our case, we assume a positive relationship between this variable and the probability of adopting adaptation strategies. Indeed, farmer organizations (OPs) are most often centers of information, training and experience sharing.

#### 3.2.3 The Source of the Data

The database used for this research is a secondary database of rural households collected in Burkina Faso as part of the Permanent Agricultural Survey (EPA). This survey is implemented by the Directorate of Foresight and Agricultural and Food Statistics (DPSAA) of the Ministry of Agriculture. The EPA is a survey that is carried out annually; it covers the 45 provinces of the Burkinabe territory according to the administrative division and allows monitoring of the national situation in terms of food security. This permanent survey aims mainly to estimate the production of the provinces and the country for each crop. It also allows forecasts of cereal harvests to establish a provisional cereal balance sheet, estimate residual farmer stocks, monitor the evolution of the food security paradigm, and evaluate the performance of the agricultural sector. It also makes it possible to collect information on the different practices developed by producers to ensure sustainable production. Thus, our empirical analysis to determine the determinants of the adoption of climate‐smart agricultural practices covers 48,159 agricultural plots held by different producers during the 2019–2020 agricultural campaign.

## 4 Presentation of Results and Interpretation

This section presents the analysis of the statistical and econometric results of the multivariate probit (MVP) model. It is structured in two parts: a statistical description of the variables, the analysis, and the interpretation of the MVP estimates.

### 4.1 Descriptive Analysis of Variables

#### 4.1.1 Dependent Variable

Table 2 shows the distribution of farmers according to the different smart agricultural practices studied. Our analyses revealed that approximately 11% of producers have adopted improved seeds, 12% have adopted water and soil conservation (CES) techniques, 21% have adopted organic manure, and 19% have adopted agroforestry as climate‐smart practices (CSAs). These results confirm the low adoption rate of CSA practices among farmers in Burkina Faso.

**TABLE 2:** Modalities of the dependent variables according to the percentage of adoption.

CSA practices	Average
Improved seeds	0.113
Water and soil conservation technology (CES)	0.118
Organic manure	0.208
Agroforestry	0.190

*Note:* *Source*: Author.

This low rate of adoption of climate‐smart agriculture (CSA) practices is consistent with the findings of Abegunde et al. (2019), Ouédraogo et al. (2019), Zakaria et al. (2020), and Shani et al. (2024), who found that, globally, the adoption of CSA practices by smallholder farmers remains very low. Furthermore, this finding is consistent with the conclusions of innovation diffusion theory, according to which time is a determining factor in the adoption of an innovation (Vishwanath and Barnett 2011).

#### 4.1.2 Explanatory Variables

The results of the statistical analysis are shown in Table 3 below.

**TABLE 3:** Characteristics of qualitative and quantitative variables.

Variables	Staff	Percentage	Average	Standard deviation	Min	Max
*Gender*						
Man	30,177	62.66	—	—	—	—
Women	17,982	37.34	—	—	—	—
Age	—	—	44.61	14.11	15	75
*Education*						
No	36,521	75.83	—	—	—	—
Yes	11,638	24.17	—	—	—	—
*Farmer's organization*						
Yes	11,413	23.7	—	—	—	—
No	36,746	76.3	—	—	—	—
*Credit*						
Yes	6624	86.25	—	—		
No	41,535	13.75	—	—	—	—
*Extension service*						
Yes	16,320	33.86	—	—	—	—
No	31,839	66.11	—	—	—	—
*Property rights*						
Yes	27,845	57.82	—	—	—	—
No	20,314	42.18	—	—	—	—
*Plot Relief*						
Plain/plateau	41,977	87.16	—	—	—	—
Lowlands	3222	6.69	—		—	—
Slope	2960	6.15	—	—	—	—
*Livestock Ownership*						
Yes	40,154	83.38		—	—	—
No	8005	16.62		—	—	—
Area	—	—	0.51	0.8	0.001	22,8841
Observations			*N* = 48,159			

*Note:* *Source*: Author.

Regarding the explanatory variables (qualitative and quantitative), we note that in the sample studied, plot managers are mostly men (62.66%). This figure shows that the majority of household heads in Africa, particularly in our context, are male. Some female value managers are widows who have not remarried. The majority of producers have no level of education (75.83%), and less than 25% have received an education. This shows that efforts still need to be made in terms of education if we want to achieve good yields because education is one of the factors that facilitates the accumulation of knowledge necessary for agricultural development. For the relationships of producers with their environment, only 23.7% belong to a farmer's organization, and 33.86% have access to extension services, which shows that less than half of the producers do not benefit from the benefits of farmers' organizations and the support of technical services, which could limit the adoption of CSA. Similarly, only 13.75% of households have access to credit, and 83.38% own livestock, which could be an important source of fertilizer owing to animal excrement. Furthermore, 57.82% have property rights over exploited land. Notably, the exploited plots consisted of plains/plateaus (87.16%), lowlands (6.69%) and slopes (6.15%). This result characterizes the nature of the soils generally encountered today in Burkina Faso, which constitutes a real obstacle to agricultural development.

With respect to the quantitative variables for the entire sample, the average age of producers is 45 years, and the maximum age is 75 years. If we look at the areas exploited, producers exploit 0.5 ha on average, and the maximum area is 22.8841 ha. This state of affairs thus characterizes the small size of agricultural holdings, which are generally family run in Burkina Faso.

### 4.2 Results of the Different Tests

Before proceeding with the estimation, we check that there is no multicollinearity between our explanatory variables. The results of the variance inflation factor (VIF) test are reported in Supporting Information for Review. The VIF values for all the coefficients associated with the explanatory variables are between 1.00 and 1.68, with an average of 1.26. In other words, for each of the coefficients associated with the explanatory variables, the VIF is less than 10 (the maximum allowed value). This indicates that there is no multicollinearity problem in the models; therefore, all explanatory variables can be retained.

### 4.3 Presentation and Interpretation of the Results of the Multivariate Probit (MVP) Estimation

In this subsection, the results of the multivariate probit (MVP) are presented and interpreted.

#### 4.3.1 Correlations Between Different CSA Practices (Complements and Substitutes)

Table 4 presents the correlation results between the different climate change adaptation strategies from the multivariate probit regression. The results obtained confirm the use of a multivariate probit model. Indeed, the likelihood ratio test for the overall correlation of the error terms of the four specified models, *χ* ^2^(6) = 478.668, *p* < 0.000, is significantly different from zero at the 1% level, thus rejecting the null hypothesis that the error terms of the four CSA models are not correlated (Asule et al. 2024; Musafiri et al. 2022). Thus, the decision to adopt a climate‐smart practice is therefore determined by that of another practice and vice versa. The results also revealed complementarities (positive sign) or substitutes (negative sign) between the CSA practices taken two by two. The correlations between (i) organic manure and CES (rho21 = 0.246, *p* < 0.01); (ii) agroforestry and CES (rho31 = 0.0657, *p* < 0.01); (iii) seeds of improved varieties and agroforestry (rho43 = 0.0355, *p* < 0.01); and (iv) improved seeds and manure (rho42 = 0.0204, *p* < 0.05) are positive and significantly different from zero. These results therefore suggest that the adoption of CES as a CSA practice is accompanied by the adoption of either organic manure or agroforestry. Additionally, the adoption of agroforestry as a CSA practice is accompanied by improved seeds; in other words, producers who adopt CES to cope with climate change necessarily adopt either agroforestry or organic manure as a complement. Similarly, producers who adopt agroforestry also adopt improved seeds, and ultimately, the adoption of improved seeds is accompanied by organic manure. This last result confirms the assertion of Wainaina et al. (2016), who highlighted in their work in Kenya, that the adoption of improved drought‐tolerant varieties may require the joint use of organic fertilizers. CSAP supplements could be attributed to the desire to improve agricultural productivity, adapt to climate change and increase incomes (Oyetunde‐Usman et al. 2021). The correlations between CES and seeds (rho41 = −0.02339, *p* < 0.05) and between agroforestry and manure (rho32 = −0.0483, *p* < 0.01) are negative and significantly different from zero. This reflects a substitution relationship between these practices. In other words, producers who adopt organic manure no longer adopt agroforestry. Similarly, those who adopt CESs no longer use improved seeds. Our results are similar to those of Ndiritu et al. (2014), Musafiri et al. (2022) who reported complements and substitutes between sustainable intensification practices among smallholder farmers in Kenya.

**TABLE 4:** Correlation coefficients of climate‐smart agricultural practices (estimated from a multivariate probit model).

Practices	Coefficients	*p*
CES and Manure (rho21)	0.246***	0.000
	(0.0089)	
Agroforestry and CES (rho31)	0.065***	0.000
	(0.0087)	
Seeds and CES (rho41)	−0.023**	0.038
	(0.1126)	
Agroforestry and Manure (rho32)	−0.048***	0.000
	(0.0081)	
Seeds and Manure (rho42)	0.0204**	0.049
	(0.0104)	
Seeds and Agroforestry (rho43)	0.035***	0.000
	(0.0101)	

*Note:* Likelihood ratio test of rho21 = rho31 = rho41 = rho32 = rho42 = rho43 = 0: *χ* ^2^(6) = 478.668 Prob > *χ* ^2^ = 0.0000. *Source*: Author; ***, **, and * are the significance thresholds at 1%, 5%, and 10%, respectively.

#### 4.3.2 Robustness of the CSA Adoption Analysis Models and

The interpretation of the econometric results requires ensuring the robustness of the analysis models. Indeed, the Wald *χ* ^2^(48) = 14426.52; *p* = 0.0000 was significant, thus confirming the validity of the analysis by the MVP model. Consequently, the null hypothesis that CSAs such as CES, agroforestry, organic manure, and seeds were independent was rejected; therefore, the hypothesis of interdependence of CSA practices was verified. The results show that the adoption of CSAs is significantly influenced by a number of socioeconomic, institutional, and biophysical factors of producers, which allows an interpretation of the estimated coefficient.

#### 4.3.3 Interpretation of the Determinants of the Adoption of CSA Practices

According to Table 5 above, the MVP results show that the adoption of CSA is significantly influenced by a number of socioeconomic, institutional, and biophysical factors of producers. Thus, we have:

**TABLE 5:** Results of the multivariate probit (MVP) estimation.

Variables	Water and soil conservation	Organic manure	Agroforestry	Improved seeds
	Coefficient (standard error)	Coefficient (Standard error)	Coefficient (Standard error)	Coefficient (Standard error)
Age	0.0046***	0.00839***	−0.0020***	−0.0036***
	(0.0005)	(0.0005)	(0.0004)	(0.0006)
Gender	0.0063	0.4645***	0.1692***	0.3360***
	(0.0206)	(0.0186)	(0.0163)	(0.0230)
Education	0.0095	−0.1186***	0.0303**	0.1103***
	(0.0184)	(0.0164)	(0.0154)	(0.0181)
Farmer's organization	−0.0205	0.1301***	0.0606***	0.3687***
	(0.0211)	(0.0187)	(0.0181)	(0.0203)
Access to credit	−0.3514***	−0.3174***	0.2225***	0.3605***
	(0.0242)	(0.0225)	(0.0226)	(0.0220)
Livestock ownership	0.0518**	0.2323***	0.0945***	0.1179***
	(0.0242)	(0.0234)	(0.0179)	(0.0285)
Extension service	0.1314***	0.0524***	0.0329**	0.1453***
	(0.0177)	(0.0155)	(0.0151)	(0.0182)
*Plot relief*				
Shallow water	0.4878***	−0.3617***	−0.3543***	0.1371***
	(0.0266)	(0.0311)	(0.0243)	(0.0317)
Slope	0.4814***	−0.0474**	0.0982***	0.0781**
	(0.0274)	(0.0278)	(0.0267)	(0.0317)
Property rights	0.2767***	0.2740***	0.1166***	0.0778***
	(0.0185)	(0.0161)	(0.0148)	(0.0188)
Surface area	0.0271***	0.0152*	1.1717***	0.1477***
	(0.0094)	(0.0080)	(0.0170)	(0.0087)
Constant	−1.7226***	−1.8264***	−0.2342***	−1.8090***
	(0.0309)	(0.0290)	(0.0234)	(0.0341)
Number of observations			***N* = 48,159**	
Likelihood ratio			**Wald *χ*** ^**2**^ (48) = 14,426,52***	

*Note:* Source: Author, based on 2019 EPA data; ***, **, * are the significance thresholds at 1%, 5%, and 10%, respectively.

##### 4.3.3.1 Age

The results show that older plot managers tend to adopt more CES and manure than younger plot managers do. These results are in agreement with those of other studies, Yabi et al. (2016) who argue that older farmers adopt innovations more easily than younger farmers do. However, this variable had a negative and significant influence at the 1% level on the adoption of improved seeds and agroforestry. This observation can be explained by the dynamism and curiosity of younger operators in the adoption of new technologies. These results corroborate those reported by Issoufou et al. (2017). In addition, this category of producers develops more of an associative spirit favorable to the acquisition of information and new knowledge on new technologies (Adekambi and Hinnou 2020). The squared age of the manager had no influence on the adoption of climate‐smart practices, which reflects a linear effect of this variable.

##### 4.3.3.2 Gender

The gender of the plot manager has a positive and significant relationship with the adoption of the four climate‐smart agricultural practices at the 1% level. When the plot manager is a man, this increases the probability of adopting improved manure, agroforestry, and seeds by 0.46, 0.17, and 0.34, respectively. These results indicate that female heads of household are disadvantaged in terms of access to productive resources and the capacity to invest in new agricultural technologies. These results confirm those of Mentire and Gecho (2017), who reported a positive correlation between gender and the adoption of climate‐smart practices.

##### 4.3.3.3 Education

This variable has a positive and significant effect on the adoption of agroforestry and improved seeds at significance levels of 5% and 1%, respectively. Our results indicate that educated farmers are more likely to adopt agroforestry and improved seeds as climate‐smart agricultural practices. When a farmer is educated, he or she is more likely to acquire and assimilate new knowledge and information in response to the problem of climate change. Education is supposed to improve an individual's management capabilities and provide him or her with several opportunities. Educated farmers, who are aware of climate change, will adopt more strategies to counter this phenomenon. Various studies have reached the same conclusion that the relationship between education and adaptation to climate change is positive (Tiwari et al. 2014; Sanga et al. 2013; Tazeze et al. 2012). However, this variable had an effect contrary to our expectations for the adoption of organic manure. This counterintuitive result could be explained, according to Faleye and Afolami (2020) in Nigeria and Negera et al. (2022) in Ethiopia, by the fact that educated people prefer office jobs to diversify their income rather than turning to food self‐sufficiency. Some educated producers are also turning to other types of fertilizers, such as chemical fertilizers, to increase their production. In the specific case of Burkina Faso, this result could be explained by the fact that educated household heads tend to allocate labor to off‐farm activities and engage in nonfarm activities with higher expected returns (Zahonogo 2002). Furthermore, Noufé et al. (2025) show that the adoption of agricultural technologies can be influenced by several interdependent factors within the decision‐making environment in which farmers operate (Danso‐Abbeam et al. 2020). For the authors, education level and income could be considered both as incentives and attractions. Indeed, farmers who are more educated and have higher incomes can easily adopt agricultural technologies, just as adoption could be a necessity for less educated farmers with low incomes in order to improve their situation.

##### 4.3.3.4 Farmer's Organization

Concerning the producer's membership in a farmer's organization, the results are positive and significant at the 1% threshold for the adoption of agroforestry and improved seeds, respectively. Indeed, an individual's membership in a farmer's organization improves the probability of adopting agroforestry and improved seeds. These results could be explained by the fact that producer groups most often constitute centers of information, extension, experience sharing, and social mutual assistance. These results are in line with our expectations and corroborate those of Mwungu et al. (2020).

##### 4.3.3.5 Access to Credit

Access to credit has a mixed effect on the adoption of smart farming practices. It is negative and significant at the 1% level for the adoption of CESs and manure and positive and significant at the 1% level for agroforestry and improved seeds. When farmers have access to credit, their level of adoption for agroforestry and improved seeds increases. Compared with agroforestry and improved seeds, access to credit has a negative effect on the adoption of CES and manure. Indeed, credit is a source of financing that increases farmers' ability to meet the transaction costs associated with adopting climate change adaptation strategies. Access to credit increases financial resources, reduces financing constraints, and allows farmers to purchase inputs. The negative relationship between the credit variable and the adoption of CES and agroforestry is contrary to our expectations but could be explained by the fact that in the context of Burkina Faso, producers often have the necessary resources to carry out these practices, for example, stones for stone barriers and animals for manure. Furthermore, the number of producers with access to credit is very low (13.75% in our case), and they tend to substitute certain climate‐smart agricultural practices with chemical fertilizers for short‐term results. Indeed, producers who have access to credit also have more access to chemical fertilizers, which they use more as fertilizers instead of sustainable techniques for restoring their soils. Similar results emerge from the work of (Nkomoki et al. 2018) in Zambia and Maré (2022) in Burkina Faso. Furthermore, this observation could be explained, on the one hand, by the credit constraints faced by producers in Burkina Faso and, on the other hand, by the fact that adoption could be a necessity for low‐income producers to improve their situation (Noufé et al. 2025).

##### 4.3.3.6 Livestock Ownership

This variable also has a positive and significant effect on the adoption of climate‐smart agricultural practices. When the number of livestock increases, the probability of adopting SWC, manure, agroforestry, and seeds increases. In our context, particularly in rural areas, some animals are used for field work and for transportation (especially horses, donkeys, and oxen). This important role played by animals gives the producer a certain flexibility in terms of his agricultural calendar. These results corroborate the conclusions of (McIntire et al. 1992). For the latter, the integration of livestock into cropping systems is one of the driving forces behind viable and sustainable integrated agriculture. Similarly, some authors, Smith et al. (1997) argue that in the context of population pressure, maintaining soil fertility and providing feed for livestock appear to be the main areas of mutual benefit. They also argue that animal ownership may be an option for increasing land productivity.

##### 4.3.3.7 Access to Extension Services

This variable has a positive and significant effect on the decision to adopt the CSA practices defined in our work. Thus, producers who had access to extension services during the growing season and/or past have a greater probability of adopting CSA practices than those who did not benefit from these services. Indeed, extension agents are responsible for transferring technologies to producers (Diallo 2019). Contacts with extension agents likely play a central role in equipping farmers with practical skills for implementing CSA practices to improve agricultural productivity and adapt to climate change. This relationship highlights the need to improve the provision of extension services to farmers, especially when climate change and technical progress are increasing at an exponential rate. Diallo (2019). On the one hand, and on the other hand, Mutua‐Mutuku et al. (2017) also estimated a positive effect of access to extension services on the adoption of CSA practices. Similarly, our results are consistent with those of Musafiri et al. (2022), who emphasized the importance of extension services in promoting the adoption of agricultural interventions.

##### 4.3.3.8 Relief of the Plot

According to some authors (Tazeze et al. 2012; Deressa et al. 2009), the adoption of climate‐sensitive agricultural practices is specific to the agroecological zone in which the plot is located. As expected, the choice of agricultural practices depends on the relief of the cultivated plot. Our results show that, compared with plots located in plain/plateau areas, the probability that a farmer opts for CES and improved seeds increases for lowland reliefs at the 1% threshold and decreases for the adoption of manure and agroforestry at the 1% threshold. Furthermore, the probability of using SWC, agroforestry, and improved seeds increases for plots located in slope areas at the 1% and 5% thresholds and decreases for the adoption of manure at the 5% threshold.

##### 4.3.3.9 Property Rights

This variable has a positive and significant effect on the adoption of smart agricultural practices at the 1% threshold. When the producer has a property right over the plot, this increases the probability of adopting CSA practices to protect against the negative effects of climate change. Indeed, holding a property right constitutes a guarantee for the owner of the plot, which encourages him to invest more in terms of sustainable agricultural practices to increase his production. In addition, land security allows access to credit, which could facilitate the adoption of new agricultural technologies, which are sometimes financially demanding. In this sense, the government should facilitate the obtaining of land titles for producers to help them integrate the fight against climate change into their production process. Our results are similar to those of Richard et al. (2022), who also showed in Burkina Faso that formal property rights positively influence the probability of the adoption of stone bunds, half‐moons, and live hedges by cereal producers in Burkina Faso.

##### 4.3.3.10 Surface Area

Agricultural area has a positive and significant effect on the adoption of CSA practices. The results indicate an increase in the likelihood of producers adopting CES, manure, agroforestry and improved seeds at the 1% threshold. These results are in line with our expectations because to increase production in large areas in a context marked by great climatic instability, producers must make large investments in terms of agricultural practices. However, Deressa et al. (2009); Hassan and Nhemachena (2008) noted that there is controversy over the relationship between plot size and adaptation to climate change.

## 5 Conclusion and Implications for Economic Policies

Agriculture remains the main subsistence activity for the majority of the Burkinabe population and the foundation of the country's economic development. However, this sector has unfortunately been disrupted in recent years by climatic hazards; this has led to a decrease in agricultural yields and, in turn, negatively impacts the food security of the population. To address this situation, efforts must be made to promote and facilitate the adoption of various climate‐smart practices. Thus, the present work aims to analyze the factors that limit or promote the adoption of climate‐smart practices among farmers in Burkina Faso. Descriptive statistics show that the adoption of CSA practices remains low among producers. To obtain the results, we used a multivariate probit model on data from a sample of 48,159 plots from the 2019 Permanent Agricultural Survey (EPA) carried out by the Ministry of Agriculture, which covers the entire national territory. The results of our estimations indicate that the level of CSA adoption varies due to differences in socioeconomic, institutional, and biophysical factors among the sampled farmers. These factors included gender, age of the plot manager, education, relief of the plot, access to extension service, access to credit, membership in a Farmer's organization, property rights, and area farmed. Correlation coefficients positive and negative correlations between practices were also established, indicating that they acted as complements and substitutes.

On the basis of these results, adequate actions that aim at adapting to climate change through the use of CSA practices could be undertaken by government agencies and rural development organizations. Therefore, we recommend that the State and these rural development organizations focus on raising awareness and training farmers on the need to adopt climate‐smart practices. Policies could promote access to credit and implement actions that facilitate access to formal land ownership documents, as when farmers have property rights, this encourages them to invest more and, in the long term, in climate‐smart practices.

## Funding

The authors did not receive any specific funding for this study.

## Ethics Statement

This article does not contain any studies with human participants or animals performed by the authors.

## Consent

All authors agree to the publication of the work.

## Conflicts of Interest

The authors declare no conflicts of interest.

## Data Availability

The data used is freely accessible via the link https://www.spcpsa.bf/download/enquete‐permanente‐agricole‐epa‐2019_2020/.
